# MRSA Knowledge, Infection-Prevention Practices, and Antibiotic Use Without Clinician Consultation Among Hajj Pilgrims Arriving in Madinah, Saudi Arabia: A Cross-Sectional Study †

**DOI:** 10.3390/tropicalmed11100270

**Published:** 2026-09-28

**Authors:** Abrar Aloufi, Mohammed Hashim Albashir, Abubaker M. Hamad, Hossameldeen Elsabaa, Abdulrhman M. Alansari, Hanin M. Abalkhail

**Affiliations:** 1 Department of Basic Medical Sciences, Al-Rayan National College of Medicine, Al-Rayan National Colleges, Al Madinah Al Munawwarah 42541, Saudi Arabia; 2 Department of General Sciences, Al-Rayan National College of Health Sciences, Al-Rayan National Colleges, Al Madinah Al Munawwarah 42541, Saudi Arabia; hm.alkhail@amc.edu.sa; 3 Department of General Sciences, Al-Rayan National College of Nursing, Al-Rayan National Colleges, Al Madinah Al Munawwarah 42541, Saudi Arabia; ab.hamad@amc.edu.sa; 4 Department of Clinical Pharmacy, Al-Rayan National College of Health Sciences, Al-Rayan National Colleges, Al Madinah Al Munawwarah 42541, Saudi Arabia; hm.elsabaa@amc.edu.sa; 5 Pharmaceutical Care Services, King Salman Medical City, Madinah Health Cluster, Medinah 42316, Saudi Arabia; amalansari@moh.gov.sa

**Keywords:** antimicrobial resistance, antibiotic use without clinician consultation, Hajj, health education, mass gathering, methicillin-resistant *Staphylococcus aureus*, MRSA, pilgrims

## Abstract

Background: Hajj is an international mass gathering in which infection prevention and antimicrobial stewardship intersect. Methicillin-resistant *Staphylococcus aureus* (MRSA) was selected as the organism-specific focus because it is a clinically important antimicrobial-resistant pathogen associated with skin and soft-tissue infections, wound care, and transmission through close contact and sharing of personal items. Hajj studies have also documented MRSA-related carriage and broader antimicrobial-resistance concerns among pilgrims and other mass-gathering populations. However, the relation between general infection awareness, MRSA-specific knowledge, and self-reported antibiotic-use behaviour remains less well described. This study assessed these three domains among Hajj pilgrims arriving in Madinah, Saudi Arabia. Methods: A questionnaire-based cross-sectional study was conducted during Hajj 1446H (2025). The study dataset comprised 342 completed questionnaires. Item-level descriptive analyses were used because no validated composite scoring algorithm was documented in the available study records. The primary exploratory outcome was an undated, self-reported history of taking antibiotics without consulting a doctor. Modified Poisson regression with robust variance estimated adjusted prevalence ratios (aPRs), with sex, age group, education, healthcare employment, and MRSA awareness entered simultaneously as prespecified covariates. Results: Among 342 respondents, 213 (62.3%) were male, 155 (45.3%) were aged 46–60 years, 181 (52.9%) reported postgraduate education, and 51 (14.9%) worked in healthcare. General awareness of crowding-related bacterial-infection risk was high (256/342, 74.9%), whereas MRSA-specific knowledge was lower: 110 (32.2%) had heard of MRSA, 137 (40.1%) identified personal-item sharing as a transmission route, and 124 (36.3%) recognised that MRSA can cause serious or fatal infection. Handwashing ≥ 3 times/day was reported by 310 respondents (90.6%), and 327 (95.6%) reported not sharing personal items. However, 143 respondents (41.8%) reported taking antibiotics without consulting a doctor. In the adjusted model, the prevalence of this outcome was higher among participants aged > 60 years than among those aged < 46 years (aPR 1.86, 95% CI 1.35–2.56) and among those who had not heard of MRSA (aPR 3.60, 95% CI 2.10–6.18) or did not know whether they had heard of MRSA (aPR 3.40, 95% CI 1.84–6.30), compared with those who had heard of MRSA. Conclusions: Among pilgrims arriving in Madinah, general infection-prevention awareness and self-reported hygiene practices coexisted with limited MRSA-specific knowledge, and a substantial proportion of respondents reported taking antibiotics without consulting a doctor. The observed associations are cross-sectional and non-causal; they neither establish the timing or appropriateness of antibiotic use nor demonstrate that an education intervention would change behaviour. They identify a hypothesis for prospective evaluation of multilingual Hajj education linking MRSA transmission, wound care, and antibiotic stewardship.

## 1. Introduction

Antimicrobial resistance (AMR) is a major global health threat. The 2024 Global Burden of Disease analysis estimated that 1.14 million deaths were directly attributable to bacterial AMR in 2021, and recent reviews continue to identify *S. aureus* among priority resistant pathogens [1,2]. MRSA is an important antimicrobial-resistant pathogen that can cause a wide range of infections, particularly skin and soft-tissue infections, and represents a significant public health concern because of its resistance to commonly used β-lactam antibiotics [3,4,5]. In mass-gathering settings such as Hajj, close physical contact, crowding, and shared environments may facilitate the transmission of infectious agents, highlighting the importance of knowledge and preventive practices related to MRSA [6,7].

Hajj is a large international mass gathering characterized by high population density, shared accommodation, extensive mobility, physical exertion, and crowding, all of which can create challenges for prevention of infection. Respiratory syndromes are frequently reported, although their measured frequency and definitions vary greatly across cohorts and settings [8,9]. Travel-health guidance therefore emphasizes personal hygiene, respiratory etiquette, vaccination where indicated, and timely clinical assessment [10]. These measures are relevant to infection prevention generally, but do not by themselves establish MRSA-specific knowledge or antibiotic-use behaviour.

*S. aureus* has been documented among Hajj pilgrims as both a colonizing organism and a cause of bacterial skin infection, while MRSA carriage has also been reported. In a cohort of 411 pilgrims during the 2004 Hajj, *S. aureus* carriage was identified in 20.6% of participants, including MRSA carriage in 1.46% [11]. Among Hajj pilgrims with pyoderma in Makkah, *S. aureus* was isolated from 65.6% of primary cases and 44.7% of secondary cases [12]. More recently, a prospective cohort of French Hajj pilgrims reported *S. aureus* detection in 14.4% before travel and 21.4% after the pilgrimage, while MRSA detection increased from 4.1% to 10.6% [13]. Together, these findings support the relevance of *S. aureus*, particularly MRSA, to Hajj-related infection prevention and health education.

Although the core Hajj rituals take place in Makkah and the surrounding holy sites, Madinah is an important setting in the broader Hajj journey, where pilgrims may be present before or after travelling to the main ritual sites. Previous studies have documented pilgrim healthcare use and health conditions in Madinah during the Hajj season, indicating that the city represents an additional setting in which pilgrims may experience health-related challenges [14,15]. The present study therefore focused on pilgrims encountered in Madinah, including those visiting before travelling to Makkah and those returning after completing Hajj. This setting provides a complementary perspective to studies conducted at the main Hajj ritual sites and allows assessment of infection-prevention awareness, MRSA-related knowledge, and self-reported antibiotic-use behaviour in pilgrims encountered outside the core ritual environment.

Microbiological studies provide an important rationale for focusing on MRSA in the Hajj setting. Reviews have documented AMR acquisition and carriage at Hajj and other mass gatherings, while consistently noting heterogeneity in study populations, settings, methods, and organisms [6,7]. A Hajj-specific scoping review identified 51 studies of antimicrobial-resistant bacteria, and a multicountry mass-gathering review found that most of its included original studies concerned religious gatherings [6,7]. In a cohort of French Hajj pilgrims, molecular MRSA detection increased from 4.1% before travel to 10.6% after Hajj [13]. These findings make MRSA a relevant education topic, but they do not measure carriage, infection, or transmission in the participants in the present questionnaire survey.

A second, separate layer concerns antibiotic knowledge and behaviour. Hajj-specific surveys and cohort studies have reported common antibiotic use and variable knowledge, but differ in questionnaire wording, setting, clinical context, and recall period [16,17]. In Saudi Arabia and the wider Middle East, community surveys and systematic reviews also document non-prescribed antibiotic use and variable understanding of appropriate use; their estimates are not directly comparable because populations and outcome definitions differ [18,19,20,21]. A recent global meta-analysis likewise reported marked heterogeneity in antibiotic use without clinician consultation estimates across studies [22].

The rationale for considering these domains together is that general infection-prevention awareness does not necessarily translate into recognition of a specific antimicrobial-resistant pathogen or into antibiotic-use behaviour. MRSA was selected because it is a clinically important resistant pathogen associated with skin and soft-tissue infection and because Hajj-related studies have documented MRSA carriage and antimicrobial-resistance concerns in pilgrims and mass gatherings [6,7,13]. Thus, MRSA-specific knowledge adds an organism-specific dimension to otherwise general infection and antibiotic-use questions. Prior microbiological work does not answer this behavioural question, and previous KAP studies do not necessarily measure organism-specific knowledge. Accordingly, this study does not claim to measure MRSA prevalence or to be the first Hajj KAP survey. It describes whether these domains were aligned or discordant in a 2025 sample of pilgrims arriving in Madinah.

The primary objective was to describe item-level MRSA knowledge, infection-prevention attitudes and practices, and self-reported antibiotic use without clinician consultation. The secondary, exploratory objective was to assess adjusted associations between selected demographic and MRSA-awareness variables and the latter outcome. No causal pathway was hypothesized: because the data are cross-sectional, all association analyses are interpreted as descriptive and hypothesis-generating.

## 2. Methods

### 2.1. Study Design and Setting

This was a questionnaire-based cross-sectional study of Hajj pilgrims arriving in Madinah Al Munawarah, Saudi Arabia, during Hajj 1446H (2025). The report follows relevant STROBE principles for transparent reporting of observational research [23]. It does not include a microbiological sampling component; there were no cultures, polymerase-chain-reaction assays, antimicrobial-susceptibility tests, or measurements of MRSA carriage or infection.

### 2.2. Participants, Recruitment, and Sample Size

The study dataset contained 342 completed participant records. Participants were Hajj pilgrims aged ≥ 18 years who were present in or visiting Madinah during the 1446H/2025 Hajj season. Participants were recruited from 22 May 2025 to 11 July 2025, including pilgrims who visited Madinah before travelling to Makkah to perform Hajj and those who visited Madinah after completing Hajj. Recruitment took place at multiple locations where pilgrims were encountered, including pilgrims’ accommodation sites, clinics around the Prophet’s Mosque, hospitals, and other pilgrim gathering areas. Members of the research team approached pilgrims at these locations and invited them to participate. Those who agreed completed the questionnaire electronically using Google Forms. Recruitment continued throughout the predefined data-collection period and was stopped on 11 July 2025 as the Hajj season ended and pilgrims departed Madinah. The planned target sample size was 500 participants. Based on an estimated Hajj population of 1,673,230 pilgrims, assuming a 95% confidence level, a 5% margin of error, and a conservative expected proportion of 50%, the minimum required sample size was approximately 384 participants and was rounded to 385. The target was therefore set at 500 to provide an additional margin for non-response and incomplete questionnaires. A total of 342 completed questionnaires were obtained. All 342 records in the study dataset were retained for descriptive and primary analyses, as shown in Figure 1. Recruitment was affected by practical field challenges, including language barriers among some pilgrims, the limited availability of many pilgrims because of worship and visits, and the need for rest between movements among different sites in Madinah. Because recruitment was non-probability based and participants were approached directly at locations where pilgrims could be encountered, participation may have been influenced by practical factors such as ease of communication, availability, and willingness to participate. Although recruitment covered a range of locations commonly used or visited by pilgrims in Madinah, the findings may provide a useful but partial picture of infection-prevention awareness, MRSA-related knowledge, and antibiotic-use behaviour among Hajj pilgrims, rather than a fully representative estimate of the broader Hajj pilgrim population. The 1,673,230 figure represents the official total number of pilgrims performing Hajj in Saudi Arabia in 1446H/2025 and was not used as a recruitment denominator or to estimate response rate [24].

### 2.3. Questionnaire and Variables

The questionnaire was developed by the research team based on a review of the relevant literature and previously published questionnaires addressing knowledge, attitudes, practices, infection prevention, and antibiotic use among Hajj pilgrims. The questionnaire was administered bilingually in Arabic and English. No formal back-translation procedure was documented. The questionnaire was reviewed by researchers before field administration and underwent institutional ethical review. Prior to the main data collection, the questionnaire was pilot-tested among 15 senior-level students and academic faculty members to assess clarity, structure, and electronic functionality across a broad age range. Based on the pilot feedback, the age item was changed from a numerical response to categorized age groups, and the information-source question was changed from a single-response to a multiple-response format. The pilot participants were not included in the final study sample. The questionnaire is therefore described as a reviewed and pilot-tested instrument rather than as a formally validated instrument. Formal psychometric validation and reliability statistics were not documented, and no composite KAP score was constructed; all results are reported at item level.

MRSA knowledge was measured at item level: having heard of MRSA; recognition of transmission through sharing personal items; recognition that crowding increases bacterial-infection risk; understanding that regular handwashing reduces MRSA transmission; and recognition that MRSA can cause serious or fatal infection. The outcome “taking antibiotics without consulting a doctor” had no documented recall period in the available study documentation. It is therefore reported as an undated, self-reported history and is not interpreted as antibiotic use during Hajj, recent use, prescribing, dispensing, misuse, or treatment appropriateness.

Nationality responses were standardized into 50 country categories and were summarized descriptively; nationality was not included as a covariate in the adjusted model.

### 2.4. Data Quality and Statistical Analysis

The study spreadsheet included 342 records and 30 original variables. One response was missing for the “visit a doctor” wound-management item. Three complete profiles were exact duplicates across the 30 original variables. Because the questionnaire did not collect unique participant identifiers, repeated participation by the same individual could not be completely excluded, and it was not possible to distinguish duplicate submissions from genuine participants with identical responses. All records were therefore retained in the primary analysis, and a sensitivity analysis excluding these profiles (n = 339) produced materially unchanged principal estimates (Supplementary Table S2). Unless otherwise specified, the denominator was 342.

Categorical variables are presented as frequencies and percentages. Multiple-response questions are presented per item and do not sum to 100%. Figure 2 displays the five knowledge items; it distinguishes the one general bacterial-infection item from MRSA-specific items, without claiming a validated knowledge score.

For all tables and text, N denotes the total number of participants in the relevant analytic sample and n denotes the number of participants in a reported category or item response. The primary exploratory model estimated adjusted prevalence ratios (aPRs) for the common binary outcome of self-reported history of antibiotic use without clinician consultation (143/342, 41.8%). We used modified Poisson regression with a log link and robust (HC0 sandwich) variance because this approach directly estimates prevalence ratios in cross-sectional studies and avoids interpreting odds ratios as risks when outcomes are common [25]. Sex, age group, education level, healthcare employment, and MRSA-awareness category were entered simultaneously because they were prespecified on study-objective and epidemiological grounds. MRSA awareness was included to examine whether the reported outcome varied by this specific knowledge measure after demographic adjustment; it was not selected by a univariable p value and is not interpreted as a causal exposure. The self-reported skin-infection item was excluded because its timing relative to the undated antibiotic-use outcome could not be established. The sole missing original response was for the wound-management item “visit a doctor”, which was neither a model covariate nor the outcome; thus the adjusted analysis included all 342 participants. The model converged successfully; model deviance was 204.10, AIC was 508.10, and predictor variance-inflation factors ranged from 1.03 to 1.96. We conducted sensitivity analyses excluding the three duplicate profiles and restricting the sample to non-healthcare workers. Associations are cross-sectional, exploratory, and non-causal; they may reflect health literacy, previous healthcare contact, access to antibiotics, clinical need, selection, reverse causality, residual confounding, or unmeasured factors. Two-sided p values are reported without adjustment for multiple comparisons. Analyses were conducted in Python 3.12 using pandas 2.2.3, SciPy 1.18.1, and statsmodels 0.15.0.

### 2.5. Ethics and Consent

The study was approved by the Institutional Review Board (IRB) of King Salman bin Abdulaziz Medical City, Madinah, Saudi Arabia (IRB No. 25-035; 12 May 2025). The study protocol and questionnaire underwent institutional ethical review before field administration. Informed consent was obtained electronically through the first page of the Google Forms questionnaire; participants had to indicate their consent before they could access the study questions. Participation was voluntary, and the questionnaire did not permit participants to proceed to the survey without providing consent.

## 3. Results

### 3.1. Participant Characteristics and Information Sources

Of 342 respondents, 213 (62.3%) were male and 129 (37.7%) were female. The largest age group was 46–60 years (155/342, 45.3%); 181 (52.9%) reported postgraduate education, and 51 (14.9%) worked in healthcare. Thirty-seven respondents (10.8%) reported a skin infection during Hajj. Physicians and health campaigns were commonly reported information sources (Table 1).

### 3.2. Knowledge, Attitudes, and Practices

General infection-risk awareness was more common than MRSA-specific knowledge. While 256 respondents (74.9%) agreed that crowding during Hajj increases bacterial-infection risk, only 110 (32.2%) had heard of MRSA. One hundred and thirty-seven (40.1%) identified sharing personal items as a route of MRSA transmission, 195 (57.0%) agreed that regular handwashing reduces MRSA transmission, and 124 (36.3%) recognised potential serious or fatal MRSA infection (Table 2).

Concern about a skin infection was reported by 190 respondents (55.6%). Most agreed that personal hygiene is more important during Hajj than usual (248/342, 72.5%) and that they try to prevent infection to protect themselves and others (262/342, 76.6%). However, 138 respondents (40.4%) agreed that antibiotics could be used without a prescription if needed.

Handwashing ≥ 3 times/day was reported by 310 respondents (90.6%), and 327 (95.6%) reported not sharing personal items. In contrast, 143 respondents (41.8%) reported taking antibiotics without consulting a doctor. When asked how they would act after a wound or boil, 287/341 (84.2%) selected visiting a doctor. The wound-management question permitted multiple responses; 112/342 (32.7%) also selected taking antibiotics without consulting a doctor (Table 3).

Exploratory adjusted associations with antibiotic use without clinician consultation.

After simultaneous adjustment for sex, age group, education, healthcare employment, and MRSA awareness, participants aged > 60 years had a 1.86-fold higher adjusted prevalence of reporting antibiotic use without clinician consultation than participants aged < 46 years (aPR 1.86, 95% CI 1.35–2.56; *p* < 0.001). Compared with respondents who had heard of MRSA, the adjusted prevalence was 3.60-fold higher among those who had not heard of MRSA (aPR 3.60, 95% CI 2.10–6.18) and 3.40-fold higher among those answering “do not know” (aPR 3.40, 95% CI 1.84–6.30; both *p* < 0.001). These are cross-sectional associations only and do not establish a causal pathway (Table 4).

Education-stratified analyses were exploratory. Education level was associated with having heard of MRSA (Pearson χ^2^ = 85.346, df = 4, *p* < 0.001), recognition that MRSA can cause serious or fatal infection (χ^2^ = 48.893, df = 2, *p* < 0.001), and reported handwashing frequency during Hajj (χ^2^ = 20.163, df = 4, *p* < 0.001). The association with reporting no sharing of personal items was also statistically significant (χ^2^ = 8.424, df = 2, *p* = 0.015), but one expected cell count was <5 and this result is interpreted cautiously. Detailed education-stratified results are provided in Supplementary Table S1. These analyses are exploratory and do not imply that education causes differences in knowledge or preventive practice.

Sensitivity analyses showed that the principal MRSA-awareness associations were stable after excluding the three exact duplicate profiles (n = 339; aPR 3.50, 95% CI 2.05–5.99 for not having heard of MRSA; aPR 3.31, 95% CI 1.79–6.12 for “do not know”) and when restricting the analysis to non-healthcare workers (n = 291; aPR 4.17, 95% CI 1.97–8.82 and aPR 4.18, 95% CI 1.85–9.43, respectively). The results are detailed in Supplementary Table S2.

## 4. Discussion

This cross-sectional study of 342 Hajj pilgrims arriving in Madinah found that general preventive awareness, organism-specific knowledge, and self-reported antibiotic-use behaviour were not consistently aligned. General awareness of crowding-related bacterial-infection risk was relatively high, and reported basic prevention practices—frequent handwashing and non-sharing of personal items—were common. In contrast, MRSA-specific knowledge was limited, and 41.8% of participants reported having taken antibiotics without consulting a doctor. The central message is therefore not that pilgrims lack all infection-prevention awareness. Rather, the findings point to specific gaps in MRSA-related knowledge alongside a substantial proportion reporting antibiotic use without clinician consultation.

The lower prevalence of MRSA-specific awareness is relevant because the Hajj evidence base contains microbiological and behavioural dimensions that should not be conflated. Hajj-specific and mass-gathering reviews document resistant bacteria, acquisition, and carriage in heterogeneous studies [6,7], and a French cohort reported increased molecular MRSA detection after Hajj [13]. International travel is also a recognized pathway for AMR movement [26]. The present study neither replicates nor contradicts these laboratory findings, because it collected no biological samples. Its contribution is different: it identifies whether pilgrims can recognize selected MRSA transmission and severity concepts. The finding that only 40.1% recognized personal-item sharing as a possible route and 36.3% recognized potentially serious or fatal infection indicates practical education targets while making no inference about individual MRSA status.

The hygiene profile should also be interpreted by domain. Studies among Hajj and Umrah pilgrims have found that participants may report frequent handwashing or other preventive practices while retaining specific knowledge gaps; reported preventive behaviour is also shaped by resource availability, context, and question wording [27,28,29]. Reviews of Hajj health education support the plausibility of targeted, culturally adapted prevention messages, but their heterogeneous designs do not establish a single best delivery strategy [30]. Because the current survey did not use a comparable validated hand-hygiene scale, it cannot compare compliance levels. It nevertheless reinforces the value of separating general hygiene practice from MRSA-specific knowledge and antibiotic stewardship.

The antibiotic-use outcome should be interpreted with caution because no recall period was specified. It had no recorded recall period and cannot be attributed to Hajj, treatment inappropriateness, access route, antibiotic class, dose, adherence, or clinical indication. Its 41.8% frequency is nonetheless pertinent to communication planning. The 2015 multi-country Hajj KAP survey reported a higher frequency of non-prescribed use, while a prospective Hajj cohort/review observed substantial antibiotic use for respiratory symptoms and identified marked variation across studies [16,17]. Saudi community surveys have likewise reported non-prescription use, and reviews from the Middle East and globally underscore the substantial heterogeneity produced by different populations, definitions, and recall periods [18,19,20,21,22]. These estimates do not form a time trend; collectively, they support a need for clear, context-sensitive stewardship communication.

The adjusted model provides a transparent exploratory assessment of the prespecified associations. Its strongest associations were with MRSA awareness: participants who had not heard of MRSA had a 3.60-fold higher adjusted prevalence of reporting antibiotics without clinician consultation relative to those who had heard of MRSA. This association does not demonstrate that MRSA education will reduce the behaviour. Awareness may mark health literacy, previous healthcare contact, underlying illness, medication access, selection, or unmeasured factors; reverse causality is also plausible. The finding therefore supports a prospective research question: whether a properly designed multilingual education intervention can improve MRSA-specific understanding and change a clearly defined antibiotic-use outcome.

The association with age > 60 years also requires caution. Older individuals may differ in care needs, medication access, previous experience of antibiotics, comorbidities, or recall; the questionnaire did not measure these factors. The finding therefore identifies a group for formative research, not a basis for clinical or policy targeting in isolation.

Physicians and health campaigns were frequently reported information sources, suggesting a practical implementation opportunity. Saudi health authorities already provide pilgrim-facing education, while evidence from Hajj/Umrah health-education studies supports testing multilingual, audience-specific materials [29,30,31]. For policy and practice, the most defensible implication of these findings is to prioritize specific educational content rather than to infer intervention effectiveness. Hajj health programs could consider integrating three messages: how MRSA can spread through close contact and shared personal items, how to manage wounds and boils safely, and why antibiotics should not be started without qualified clinical advice. Such messages could be incorporated into existing multilingual pilgrim education and delivered through physicians, health campaigns, and other established channels. The present data cannot determine the most effective channel, language, or causal effect of any message; these questions require prospective evaluation.

### Strengths and Limitations

This study has several strengths. The manuscript was reconstructed from the study dataset rather than from a prior narrative alone. It reports item-level results instead of constructing an unvalidated KAP score, documents the duplicate and missing-data findings, distinguishes the setting accurately as pilgrims arriving in Madinah, and uses an exploratory model with adjusted prevalence ratios and robust confidence intervals. The two figures add scientific value by making the data inclusion and knowledge gap visible without overstating certainty.

Key limitations include non-probability recruitment without a recruitment log, which prevented estimation of the number approached, refusals, or response rate and limits assessment of selection bias and generalisability. The high educational attainment of the sample may have contributed to greater infection-prevention awareness, reported hygiene practices, and MRSA knowledge than might be observed in a more educationally diverse Hajj population. Because the questionnaire was available only in Arabic and English, pilgrims who were not sufficiently proficient in either language may have been excluded or underrepresented, potentially introducing selection bias and limiting generalisability. In addition, because recruitment was conducted in Madinah, pilgrims who did not visit Madinah, including some domestic and regional pilgrims, may have been underrepresented. This should be considered when interpreting the findings and their generalisability to the broader Hajj pilgrim population. Outcomes were self-reported, with possible recall and social-desirability bias, and the antibiotic-use item lacked a defined timeframe and therefore cannot be interpreted as use during Hajj. The questionnaire was reviewed and pilot-tested among 15 senior-level students and academic faculty members, but formal psychometric validation and reliability statistics were not documented. Three exact response profiles were duplicated, although no unique identifier existed to justify exclusion from the primary analysis; sensitivity analyses were therefore performed. Finally, the cross-sectional design limits causal interpretation, and the study included no laboratory confirmation of MRSA.

## 5. Conclusions

Among Hajj pilgrims arriving in Madinah, general awareness of infection prevention and self-reported hygiene practices were relatively high, whereas MRSA-specific knowledge was limited; a substantial proportion of respondents reported taking antibiotics without consulting a doctor. The observed association between MRSA awareness and reported antibiotic use is cross-sectional and hypothesis-generating rather than causal, and it should not be interpreted as evidence that MRSA education would change antibiotic-use behaviour. The practical contribution of this study is to identify specific MRSA knowledge and antibiotic-stewardship content that could be incorporated into multilingual Hajj health education and tested prospectively. Future studies should use a documented recruitment approach, a fully described and formally evaluated questionnaire, a defined medication-use recall period, and—where appropriate—microbiological outcomes.

## Figures and Tables

**Figure 1:**
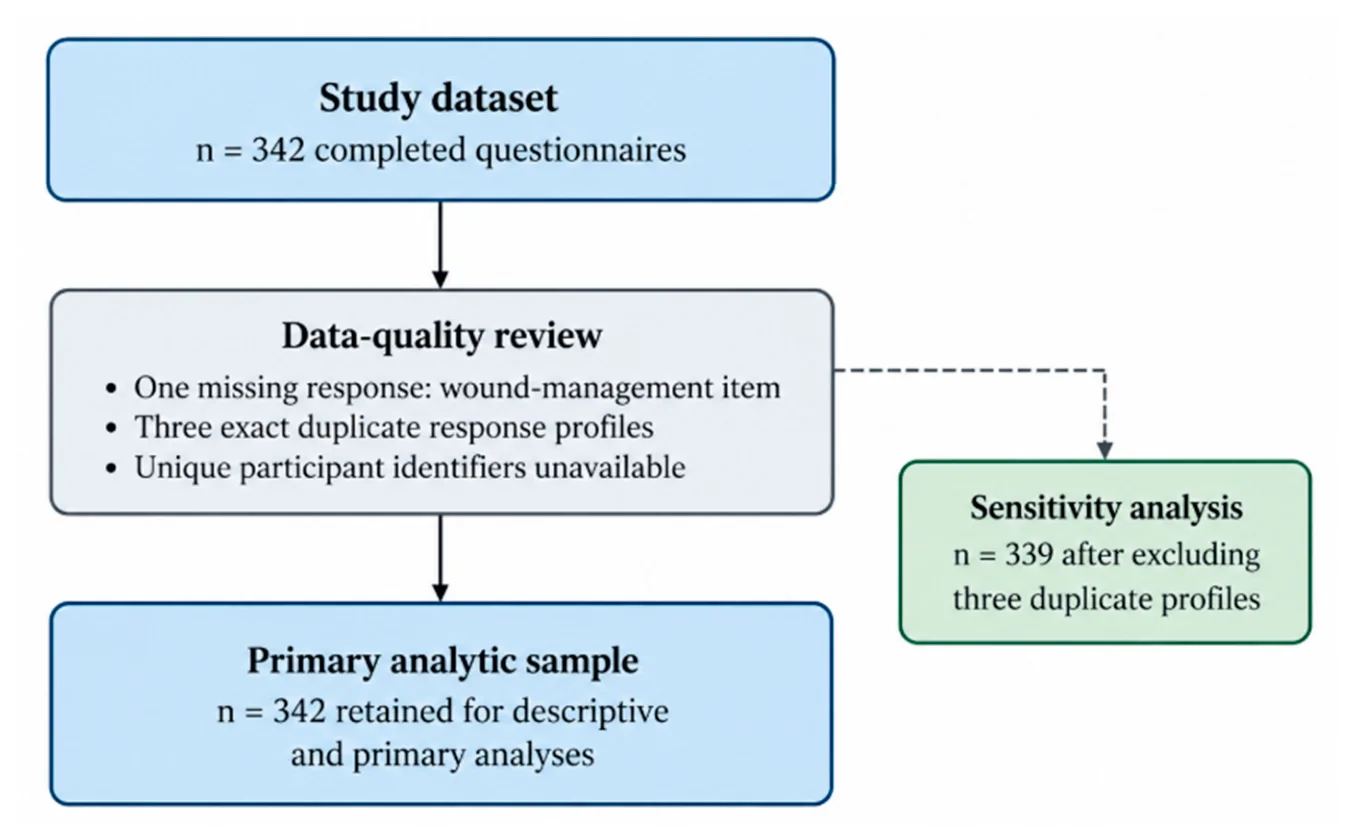
Data-quality and analysis-inclusion flow. All 342 records in the study dataset were retained for descriptive and primary analyses. Three identical response profiles were retained because unique participant identifiers were unavailable and duplicate enrolment could not be verified. This figure is not a recruitment flow; the number approached, recruitment denominator, eligibility-screening information, and response rate could not be reliably determined.

**Figure 2:**
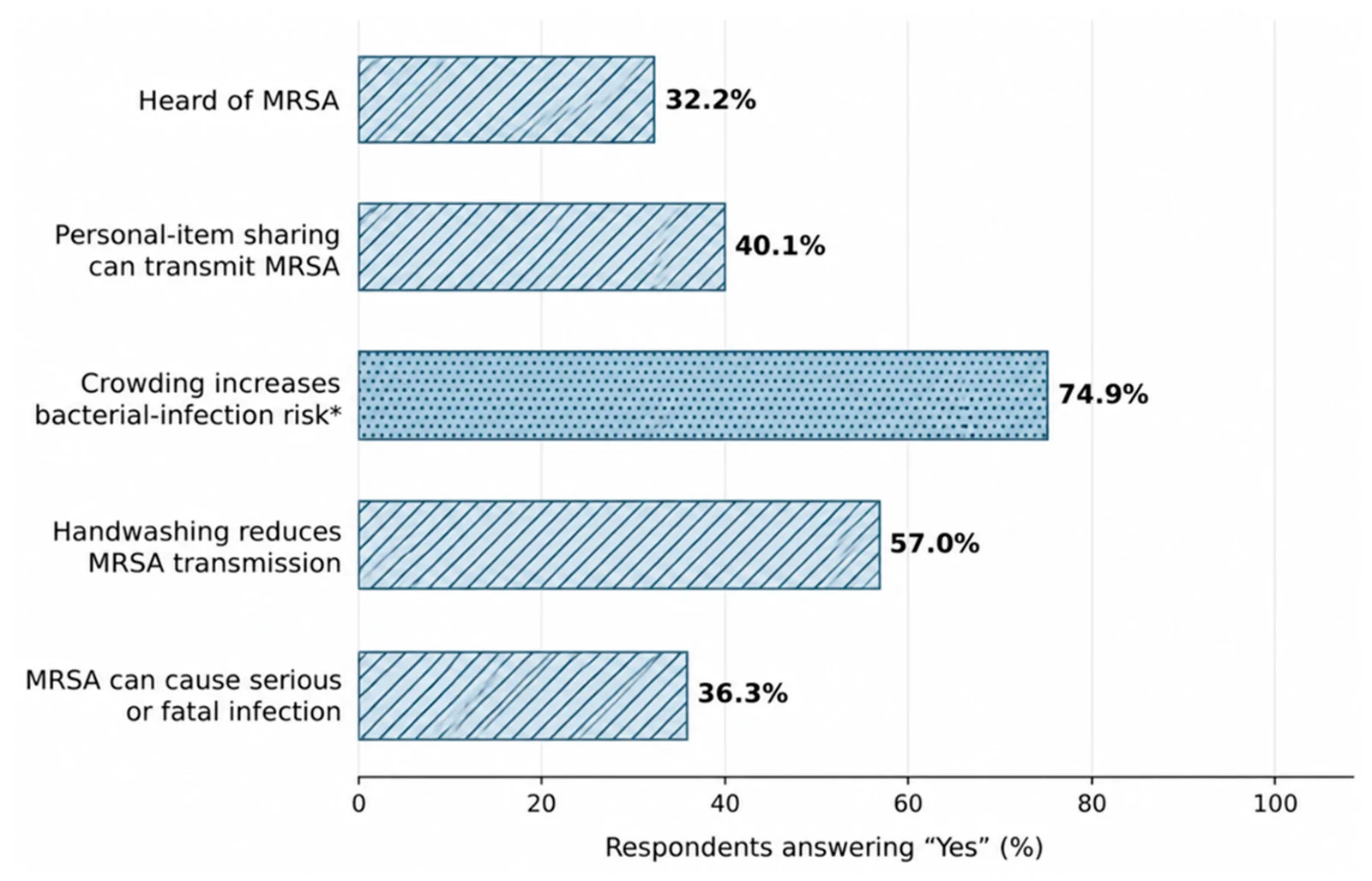
Item-level MRSA knowledge profile (N = 342). Direct percentage labels and distinct hatch patterns are used for all bars. The crowding item concerns general bacterial-infection risk; all other items concern MRSA-specific knowledge. * Refers to the general bacterial-infection risk item, not an MRSA-specific knowledge item.

**Table 1:** Participant characteristics and reported infection-information sources (N = 342).

Characteristic	n	%
Gender		
Male	213	62.3
Female	129	37.7
Age group		
<30 years	16	4.7
30–45 years	131	38.3
46–60 years	155	45.3
>60 years	40	11.7
Education level		
Illiterate	19	5.6
Primary	21	6.1
Secondary	32	9.4
University	89	26.0
Postgraduate	181	52.9
Healthcare worker	51	14.9
Self-reported skin infection during Hajj	37	10.8
Reported information source †		
Physician	259	75.7
Health campaign	195	57.0
Internet/social media	124	36.3
Friends/family	117	34.2
Other source	9	2.6

† Multiple responses were permitted.

**Table 2:** MRSA knowledge and infection-prevention attitudes (N = 342).

Item and Response	n	%
Heard of MRSA		
Yes	110	32.2
No	186	54.4
Do not know	46	13.5
MRSA can be transmitted by sharing personal items		
Yes	137	40.1
No	21	6.1
Do not know	184	53.8
Crowding increases bacterial-infection risk		
Yes	256	74.9
No	8	2.3
Do not know	78	22.8
Regular handwashing reduces MRSA transmission		
Yes	195	57.0
No	4	1.2
Do not know	143	41.8
MRSA can cause serious or fatal infection		
Yes	124	36.3
Do not know	218	63.7
Personal hygiene during Hajj is more important than usual		
Agree	248	72.5
Neutral	65	19.0
Disagree	29	8.5
Antibiotics can be used without a prescription if needed		
Agree	138	40.4
Neutral	71	20.8
Disagree	133	38.9
I try to prevent infections to protect myself and others		
Agree	262	76.6
Neutral	64	18.7
Disagree	16	4.7

**Table 3:** Self-reported hygiene, antibiotic-use, and wound-management practices.

Practice and Response	n/N	%
Handwashing frequency during Hajj		
>5 times/day	233/342	68.1
3–5 times/day	77/342	22.5
Rarely	32/342	9.4
**Carries hand sanitizer while moving**		
Always	82/342	24.0
Sometimes	119/342	34.8
No	141/342	41.2
Does not share personal items	327/342	95.6
Has taken antibiotics without consulting a doctor	143/342	41.8
**If a wound or boil develops †**		
Visit a doctor	287/341	84.2
Use disinfectant	132/342	38.6
Ignore it	30/342	8.8
Take antibiotics without consulting a doctor	112/342	32.7

† Multiple responses were permitted; one response was missing for the “visit a doctor” item.

**Table 4:** Exploratory adjusted associations with self-reported antibiotic use without clinician consultation (N = 342).

Association	aPR	95% CI	*p* Value
Male (vs. female)	1.28	0.98–1.68	0.069
Age 46–60 (vs. <46)	1.29	0.96–1.72	0.087
Age > 60 (vs. <46)	1.86	1.35–2.56	<0.001
University (vs. secondary/lower)	1.04	0.77–1.39	0.812
Postgraduate (vs. secondary/lower)	0.88	0.64–1.22	0.460
Healthcare worker (vs. non-healthcare)	1.42	0.85–2.37	0.184
Not heard of MRSA (vs. heard of MRSA)	3.60	2.10–6.18	<0.001
Do not know (vs. heard of MRSA)	3.40	1.84–6.30	<0.001

Model: modified Poisson regression with robust variance. The outcome was self-reported history of taking antibiotics without consulting a doctor. All covariates listed were included simultaneously. Reference groups are shown in parentheses. The model is exploratory and cross-sectional; aPRs are associations, not risk ratios or causal effects.

## Data Availability

The data presented in this study are available from the corresponding author upon reasonable request and subject to applicable ethical and privacy restrictions.

## Supplementary Materials

The following supporting information can be downloaded at: https://www.mdpi.com/article/10.3390/tropicalmed11100270/s1, File S1: Original Arabic–English Questionnaire; Supplementary Table S1: Education-Stratified Exploratory Analyses; Supplementary Table S2: Sensitivity Analyses and Model Diagnostic Summary.
