# Supplementary material for: MRSA Knowledge, Infection-Prevention Practices, and Antibiotic Use Without Clinician Consultation Among Hajj Pilgrims Arriving in Madinah, Saudi Arabia: A Cross-Sectional Study
Source: Trop Med Infect Dis. 2026 Sep 28;11(10):270. doi: 10.3390/tropicalmed11100270 (PMC13709939; doi:10.3390/tropicalmed11100270)
Supplement: Supplementary file 1 [file tropicalmed-11-00270-s001.zip › tropicalmed-4534523-supplementary.pdf]

Supplementary Materials

Manuscript ID: tropicalmed-4534523

MRSA Knowledge, Infection-Prevention Practices, and Antibiotic Use Without Clinician Consultation Among Hajj Pilgrims Arriving in Madinah, Saudi Arabia: A Cross-Sectional Study

Supplementary File S1. Original Arabic–English Questionnaire

The questionnaire below reproduces the original bilingual Google Forms instrument used for the study, including the informed-consent text and the question structure used for data collection. The item wording is retained as administered.

Section 1. Informed Consent

الموافقة على المشاركة في الدراسة

أنت مدعو للمشاركة في دراسة بحثية تهدف إلى تقييم معرفتك ومواقفك وممارساتك المتعلقة بالوقاية من عدوى المكورات العنقودية الذهبية المقاومة للميثيسيلين (MRSA) خلال موسم الحج. تهدف هذه الدراسة إلى تحسين التوعية الصحية والوقاية من العدوى في المستقبل.

مشاركتك في هذا الاستبيان اختيارية تمامًا، ويمكنك الانسحاب في أي وقت دون أي تبعات. جميع البيانات التي يتم جمعها ستُستخدم لأغراض البحث فقط، وستُعامل بسرية تامة ولن يتم التعرف على هويتك بأي شكل.

بإكمال هذا الاستبيان، فإنك توافق على المشاركة في هذه الدراسة طوعًا.

You are invited to participate in a research study aimed at assessing your knowledge, attitudes, and practices regarding the prevention of Methicillin-Resistant Staphylococcus aureus (MRSA) infections during the Hajj season. The goal of this study is to improve public health awareness and infection prevention strategies.

Your participation in this survey is entirely voluntary. You may skip any question or withdraw from the study at any time without any consequences. All data collected will be used for research purposes only, and your responses will remain strictly confidential and anonymous.

By completing this questionnaire, you are giving your informed consent to participate in the study voluntarily.

Section 2. KAP Questionnaire

No.	Question / Item	Response format / options
1	Gender / الجنس	Required categorical response.
2	Age / العمر	Required categorical response.
3	Nationality / الجنسية	Required country response.
4	Education level / المستوى التعليمي	Required categorical response.
5	Do you work in the healthcare field? / هل تعمل في المجال الصحي؟	Required response: Yes / No.
6	Have you had a skin infection during Hajj? / هل أصبت بعدوى جلدية أثناء الحج؟	Required response: Yes / No.
7	What are your sources of information about infection and antibiotics? (Check all that apply) / ما هي مصادر معلوماتك عن العدوى والمضادات الحيوية؟ (اختر كل ما ينطبق)	Multiple responses permitted: Internet/Social Media; Physician; Health Campaigns; Friends/Family; Other source.
8	I have heard of MRSA (Methicillin-Resistant Staphylococcus aureus) / سمعت سابقًا عن بكتيريا MRSA المقاومة للمضادات الحيوية	Yes / No / Don't know.
9	MRSA can be transmitted through sharing personal items (e.g., towels, razors) / يمكن أن تنتقل MRSA عبر مشاركة الأدوات الشخصية (مثل المناشف، موس الحلاقة)	Yes / No / Don't know.
10	Overcrowding during Hajj increases the risk of bacterial infections / الزحام أثناء الحج يزيد من خطر العدوى البكتيرية	Yes / No / Don't know.
11	Regular handwashing reduces MRSA transmission / غسل اليدين بانتظام يقلل من انتشار MRSA	Yes / No / Don't know.
12	Some MRSA strains can cause serious or fatal infections / قد تسبب بعض سلالات MRSA عدوى خطيرة أو مميتة	Yes / Don't know.
13	I am worried about getting a skin infection during Hajj / أشعر بالقلق من خطر الإصابة بعدوى جلدية أثناء الحج	Agree / Neutral / Disagree.
14	Personal hygiene during Hajj is more important than usual / أعتقد أن النظافة الشخصية خلال الحج	Agree / Neutral / Disagree.

No.	Question / Item	Response format / options
	أهم من الأيام العادية	
15	Antibiotics can be used without a prescription if needed / يمكن استخدام المضادات الحيوية دون وصفة طبية إذا لزم الأمر	Agree / Neutral / Disagree.
16	I try to prevent infections to protect myself and others / أحرص على الوقاية من العدوى لحماية نفسي والآخرين	Agree / Neutral / Disagree.
17	How many times do you wash your hands daily during Hajj? / كم مرة تغسل يديك يوميًا أثناء الحج؟	More than 5 times / 3–5 times / Rarely.
18	Do you carry hand sanitizer while moving? / هل تحمل معقم يدين أثناء التنقل؟	Always / Sometimes / No.
19	Have you shared personal items (towel, toothbrush) with others? / هل شاركت أدواتك الشخصية (منشفة، فرشاة أسنان) مع آخرين؟	Yes / No.
20	Have you taken antibiotics without consulting a doctor? / هل استخدمت المضادات الحيوية دون استشارة طبيب؟	Yes / No.
21	If you develop a wound or boils, what action do you take? / إذا ظهر لديك جرح أو دمامل، ما إجراءاتك؟	Multiple responses permitted: Visit a doctor; Use disinfectant; Ignore it; Take antibiotics without consulting a doctor.
22	Researcher name / اسم الباحث/ـة	Required field.

Note: The wound-management item permitted multiple responses, and one response was missing for "Visit a doctor" in the study dataset. The antibiotic-use outcome item did not specify a recall period.

Supplementary Table S1. Education-Stratified Exploratory Analyses

Results are presented as n (%) within each education stratum. For these exploratory analyses, education was grouped as secondary/lower (illiterate, primary, and secondary), university, and postgraduate, consistent with the grouping used for the reported chi-square analyses in the manuscript.

S1a. "I have heard of MRSA" by education level

Education level (n)	Yes	No	Don't know
Secondary/lower (n=72)	17 (23.6%)	34 (47.2%)	21 (29.2%)
University (n=89)	2 (2.2%)	69 (77.5%)	18 (20.2%)
Postgraduate (n=181)	91 (50.3%)	83 (45.9%)	7 (3.9%)
Overall (N=342)	110 (32.2%)	186 (54.4%)	46 (13.5%)

Pearson χ^2=85.346, df=4, p=<0.001.

S1b. Recognition that MRSA can cause serious or fatal infection by education level

Education level (n)	Yes	Don't know
Secondary/lower (n=72)	22 (30.6%)	50 (69.4%)
University (n=89)	8 (9.0%)	81 (91.0%)
Postgraduate (n=181)	94 (51.9%)	87 (48.1%)
Overall (N=342)	124 (36.3%)	218 (63.7%)

Pearson χ^2=48.893, df=2, p=<0.001.

S1c. Handwashing frequency during Hajj by education level

Education level (n)	More than 5 times	3–5 times	Rarely
Secondary/lower (n=72)	46 (63.9%)	16 (22.2%)	10 (13.9%)
University (n=89)	48 (53.9%)	26 (29.2%)	15 (16.9%)
Postgraduate (n=181)	139 (76.8%)	35 (19.3%)	7 (3.9%)
Overall (N=342)	233 (68.1%)	77 (22.5%)	32 (9.4%)

Pearson χ^2=20.163, df=4, p=<0.001.

S1d. Reporting no sharing of personal items by education level

Education level (n)	Yes	No
Secondary/lower (n=72)	7 (9.7%)	65 (90.3%)
University (n=89)	5 (5.6%)	84 (94.4%)
Postgraduate (n=181)	3 (1.7%)	178 (98.3%)
Overall (N=342)	15 (4.4%)	327 (95.6%)

Pearson χ^2=8.424, df=2, p=0.015.

Note: The personal-item-sharing analysis had one expected cell count <5 (minimum expected count=3.16) and was interpreted cautiously in the manuscript.

Supplementary Table S2. Sensitivity Analyses and Model Diagnostic Summary

Analysis	Analytic sample	Contrast	aPR	95% CI	p value
Primary analysis	N=342	Not heard of MRSA vs heard of MRSA	3.60	2.10–6.18	<0.001
Primary analysis	N=342	Do not know vs heard of MRSA	3.40	1.84–6.30	<0.001
Exclude three exact duplicate profiles	n=339	Not heard of MRSA vs heard of MRSA	3.50	2.05–5.99	—
Exclude three exact duplicate profiles	n=339	Do not know vs heard of MRSA	3.31	1.79–6.12	—
Non-healthcare workers only	n=291	Not heard of MRSA vs heard of MRSA	4.17	1.97–8.82	—
Non-healthcare workers only	n=291	Do not know vs heard of MRSA	4.18	1.85–9.43	—

Model diagnostic summary

The primary model used modified Poisson regression with a log link and robust (HC0 sandwich) variance. All covariates were entered simultaneously. The model converged successfully; model deviance was 204.10; AIC was 508.10; and predictor variance-inflation factors ranged from 1.03 to 1.96. The sole missing original response was for the wound-management item “visit a doctor”; it was neither the outcome nor a model covariate, so the adjusted primary analysis included all 342 participants.

Interpretation: The sensitivity estimates were materially similar to the primary estimates. All analyses are cross-sectional and exploratory and do not establish causality.

Abbreviations: aPR, adjusted prevalence ratio; CI, confidence interval; MRSA, methicillin-resistant Staphylococcus aureus.
